# Experimental manipulation of a signal trait reveals complex phenotype-behaviour coordination

**DOI:** 10.1038/s41598-018-33948-0

**Published:** 2018-10-19

**Authors:** Iris I. Levin, Bailey K. Fosdick, Toshi Tsunekage, Matthew A. Aberle, Christine M. Bergeon Burns, Amanda K. Hund, Rebecca J. Safran

**Affiliations:** 1 Department of Ecology and Evolutionary Biology, University of Colorado, Boulder, CO 80309 USA; 2 Department of Biology, Agnes Scott College, Decatur, GA 30030 USA; 3 Department of Statistics, Colorado State University, Fort Collins, CO 80523 USA; 4 Department of Biological Sciences, Virginia Tech, Blacksburg, VA 24061 USA; 5 Center for the Integrative Study of Animal Behavior, Indiana University, Bloomington, IN 47405 USA

## Abstract

Animals use morphological signals such as ornamental traits or weaponry to mediate social interactions, and the extent of signal trait elaboration is often positively associated with reproductive success. By demonstrating relationships between signal traits and fitness, researchers often make inferences about how behaviour operates to shape those outcomes. However, detailed information about fine-scale individual behaviour, and its physiological basis, can be difficult to obtain. Here we show that experimental manipulations to exaggerate a signal trait (plumage colour) and concomitant changes in testosterone and stress-induced corticosterone levels altered social interactivity between manipulated males and their social mates. On average, darkened males did not have higher levels of interactivity than unmanipulated males; however, males who experienced a greater shift in colour (pale to dark), a larger, positive change in testosterone levels, and a dampened stress-induced corticosterone response had a larger increase in the number of interactions with their social mate post-manipulation compared to pre-manipulation. This work provides new insights into the integration and real-time flexibility of multivariate phenotypes and direct evidence for the role of social interactions in pair bond maintenance.

## Introduction

The need to react rapidly to a variety of social stimuli requires a highly coordinated and integrated phenotype. Research has focused on how animals react to environmental stressors, including extreme weather events^1^, food shortages^2,3^, and increased threats from predation^2,4^; however, individuals also face additional challenges within their social environment, including competition for mates. In this context, animals have evolved signal traits to mediate social interactions with conspecifics, such as advertising fighting^5^ and foraging^6^ ability. These signal traits are often linked with suites of physiological, behavioural, and morphological traits (an ‘integrated phenotype’^7^), which enable appropriate responses to new challenges and opportunities. Despite considerable research on multivariate phenotypes, fundamental puzzles remain about how individuals appropriately coordinate their signal traits and behaviour within an often dynamic social context^8^. One hypothesis about how signal traits influence social interactions is that their information content is contingent upon social feedback which, in turn, affects aspects of physiology (e.g., hormones) and helps coordinate appropriate behaviour as the social context changes^8–10^. While we know that signal traits can affect an individual’s social environment^11^ and reproductive success^12–15^, and that changes in the social environment can rapidly induce responses in circulating hormones^16,17^, we know relatively less about whether and how all of these factors are associated with one another.

This research focuses on a two-year social network experiment in a population of North American barn swallows (*Hirundo rustica erythrogaster*), songbirds that breed socially. Ventral plumage colour, a melanin-based trait, is an important signal trait in *H. r. erythrogaster*; females preferentially allocate paternity to males with experimentally darkened ventral plumage^15,18^. Darker colouration is also linked to the hormonal state of the individual. Naturally darker males have higher circulating levels of testosterone compared to paler males, and males who had their ventral plumage experimentally darkened had elevated testosterone levels one week after the manipulation compared to unmanipulated males^9^. This provides direct evidence for a bidirectional relationship between signal traits and physiology, and indirectly suggests that the increase in testosterone in darkened males is the result of altered behavioural interactions in response to the manipulated phenotype^19^. Further, a separate correlational study using proximity tags to track social interactions revealed that females are more interactive with naturally dark males with stronger stress-induced corticosterone (CORT) responses^20^. Collectively, these correlational findings suggest that males with the most exaggerated signal traits have more interactions with females and as a response, have higher androgen levels, stress reactivity, and reproductive success.

To directly examine the role of social interactions in mate selection behaviour and phenotype integration, we investigated changes in social interactivity (measured via proximity loggers^21^) in response to experimental manipulation of male ventral plumage colour and associated natural changes in circulating testosterone levels and stress-induced CORT levels. If male signal traits are causally associated with reproductive success through increased female interactions, we predicted that males with experimentally darkened ventral plumage would receive more attention from females and therefore an increase in the number of close-proximity interactions with females. Additionally, we predicted an increase in circulating levels of both testosterone and stress-induced CORT in experimentally darkened males. In addition to testing for a treatment effect corresponding to the plumage colour manipulation, we examined whether the degree of plumage colour change was related to changes in interactivity and hormone levels.

## Methods

### Experimental design

This experiment was conducted at a large barn swallow breeding site consisting of 29–31 breeding pairs in Boulder County, CO (40°09′44.3″N, 105°11′58.1″W) between June 11–23 2015, and June 12–25 2016. Encounternet proximity loggers^20,21^ were worn by 58/59 (98%) (2015) and 56/62 (90%) (2016) of the birds for the duration of the experiment. To synchronize the collection of social interactivity data via proximity loggers, birds had to be tagged within one to two days at the start of the experiment, because the tags were programmed to turn on a few days later. Therefore, we were unable to tag 100% of the population. Recordings of close-range (≤10 cm)^21^ interactions were used to construct social networks and calculate male-female node strength (the sum of all interactions between a focal male and all females), a male’s interactivity outside the pair bond (male-female node strength excluding interactions with his social mate), and a male’s interactivity with his social mate. Briefly, these proximity tags both transmit and receive unique identification codes and can thus record social interactions with other individuals in the population. We cannot determine the direction of the interactions from the proximity data. We define interactivity as the frequency of close proximity interactions between a focal male and other birds (e.g., his social mate, other males or other females). Birds were fitted with proximity loggers three days before the loggers began collecting data. On the date they were tagged, birds were blood sampled (up to 100 μL from the brachial vein) after a 15-minute standardized restraint^22^ to measure the strength of the response to stress, and feathers were plucked from four regions of the ventral surface (throat, breast, belly, vent) for quantification of colour via spectrophotometry^15^. Sampling was done between 4–10 am, with all birds caught and tagged on one morning. Birds were banded with a USGS metal band and a unique combination of colour bands, were weighed, and their right wing and both tail streamers (outermost elongated tail feathers) were measured. On the day prior to interactivity data collection, first-clutch eggs were collected from all nests. If a nest reached two days prior to hatch date before the egg collection date, the eggs were collected early and replaced with fake eggs created to mimic the weight and appearance of barn swallow eggs. Eggs were collected for DNA extraction and paternity analyses as a record of female mating decisions prior to the plumage manipulation^15^.

Proximity data were collected from 6–9 am for two days (pre-manipulation social interactivity), after which the tags remained in sleep mode for three days. We collected proximity data during these hours because the birds are most active during this time of day. We recorded activity for a three-hour period across consecutive days in order to get replicated social interaction information during the same time period. A recording period of three hours was chosen to increase battery life over the course of several days of tag use. On the night of the last day of pre-manipulation social data collection, a random half of the males were caught on or near the nest. The ventral plumage of these males (2015: n = 14, 2016: n = 15) was experimentally darkened with a non-toxic art marker (PrismaColor Light Walnut #3507)^9,10,15,18^ and breast feathers were plucked after the application of colour to determine the degree of colour change post-manipulation compared to pre-manipulation. Two of the same males were randomly assigned to the darkened treatment in both years. Previous use of this plumage manipulation treatment and a sham control (application of a clear marker) indicates no differences between males in an unmanipulated control group (as we use here) compared to the sham control group^15^. As such, and because of time and sample size limitations associated with the use of tags to track individual interactions, we used two treatment groups in this study: a plumage enhancement and an unmanipulated control group. Three days after the plumage manipulation, the tags turned back on and collected proximity data from 6–9 am (post-manipulation social interactivity) until the batteries on the tags died (typically two or more sampling days). Most birds (2015: 95%, 2016: 93%) were re-captured on the morning of the fourth day of post-manipulation data collection for blood sampling, weighing, and to remove the proximity logger. The remaining individuals (2015: n = 3, 2016: n = 4) were recaptured over the next few days. Females were beginning to lay eggs on the date of recapture. The resulting nestlings were blood sampled twelve days after hatching, and six microsatellite markers^22^ were used to assign paternity of offspring, including eggs collected just prior to the experiment. Research protocols were approved by the University of Colorado’s IACUC (permit no. 1303.02) and all experiments were performed in accordance with relevant guidelines and regulations.

### Changes in social interactivity

In addition to comparing the levels of interactivity of males in both treatment groups (experimental manipulation of colour and no manipulation of colour) pre- and post-manipulation, we also compared within-individual changes as a function of plumage colour changes for males within the colour manipulation group. This enables a powerful, paired design comparison to analyse whether and how changes in an individual’s phenotype have concomitant changes in other aspects of the integrated phenotype. Social networks were constructed from pairwise, close-proximity interactions between tagged individuals^21^ and changes (post-manipulation minus pre-manipulation) in interactivity were calculated, including changes in male-male, male-female, and male-social mate interactivity, as well as interactivity between males and females other than the social mate. Individuals were included in the analysis if they had complete social data (two days pre- and post-manipulation, including complete data for their social mate) and hormone data.

### Plumage colour analysis

Feathers were stored in the dark in envelopes until they were measured. Feathers were overlaid and taped onto a white index card to closely resemble their appearance on the bird^15^ and colour was assessed using an Ocean Optics USB4000 spectrometer (Dunedin, FL) and a pulsed xenon light (PX-2, Ocean Optics). The probe was held perpendicular to the feathers at a set distance from the feather surface so that 2.5 mm of the feather sample was illuminated and measured. Spectral data, assessed between 300–700 nm, were generated relative to a white standard (Ocean Optics WS-1) and a dark standard (all light excluded) using SpectraSuite v.3.0.151 (Ocean Optics). Each feather region was analysed three times using 20 scans per measurement and the mean was used for further colour processing, which was done using the R package pavo^23^. We quantified three traditional axes of colour: brightness, hue, and chroma, which are all highly correlated within plumage patch^24^. Change in plumage colour for experimentally darkened males was calculated as the difference in average breast feather brightness between the spectral data from the initial natural ventral plumage colour and breast feathers collected immediately post-manipulation. Average brightness is the amount of light, regardless of wavelength, reflected from an object^25^ and ranges from white (reflects the most light) to black (reflects the least light). Therefore, a bird with darker ventral plumage has a lower brightness value than a paler bird. For more intuitive results, we used the inverse of change in breast feather brightness (post-manipulation brightness – pre-manipulation brightness) such that a larger change in brightness corresponded to a greater shift from pale to dark. We compared average brightness to colour metrics quantified in tetrahedral colour space^26^, which estimates the relative stimulation of cone types in the eyes of passerine birds. This method produced two measures that correspond to hue (theta, phi) and one measure that corresponds to saturation (r), that were moderately to highly correlated with our measure of plumage colour, average breast brightness (average breast brightness – theta: r = −0.85, average breast brightness – phi: r = −0.80, average breast brightness – r achieved: −0.60). Thus, our measure of colour, average breast brightness, is straightforward to interpret biologically, variable between males, repeatable^27^, and correlated with additional metrics obtained via models of avian vision.

### Corticosterone assay

Stress-induced CORT levels were quantified for most males and females using an assay optimized for barn swallows^10,22^. There is evidence in this population that stress-induced CORT and baseline CORT are correlated and repeatable for individuals across different life history stages within the breeding season^28^. Because we had previously found that males with higher stress-induced CORT were more interactive with females, we chose to sample stress-induced CORT instead of baseline CORT, as we were unable to sample both for this experiment. Our stress-induced CORT protocol involved a 15-minute standardized restraint used previously in barn swallows^20,22,29^. With multiple birds caught at similar times, some birds were sampled after a slightly longer restraint. Mean time to bleed for males in our analyses was 19.67 minutes (±4.30 S.D.) pre-manipulation and 18.30 minutes (±7.56 S.D.) post-manipulation. There was no relationship between time to bleed and CORT or testosterone concentrations pre- or post-manipulation (pre-manipulation CORT ~ time to bleed: β = −4.21, r^2^ = 0.33, p = 0.08, post-manipulation CORT ~ time to bleed: β = 1.059, r^2^ = −0.08, p = 0.53, pre-manipulation T ~ time to bleed: β = −0.01, r^2^ = 0.13, p = 0.78, post-manipulation T ~ time to bleed: β = 0.01, r^2^ = −0.09, p = 0.64).

Stress-induced CORT was assayed pre- and post-manipulation using an enzyme immunoassay kit (Enzo Life Science, Plymouth Meeting, PA, ADI-900-097). Pre- and post-manipulation samples for each individual were always run on the same plate along with a six-dilution standard curve run in triplicate. Samples were run in duplicate; pre-manipulation intra-assay coefficient of variation was 10.1% and post-manipulation coefficient of variation was 10.7% Inter-assay variation was 4.53%. In cases where plasma volume was low, we prioritized assaying testosterone over CORT. As with social interactivity data, we analysed changes in stress-induced CORT levels (post-manipulation levels – pre-manipulation levels).

### Testosterone assay

Testosterone assays were performed at the Indiana University’s Center for the Integrative Study of Animal Behavior using a protocol optimized for a variety of avian species, including barn swallows^10,30^. In brief, this involved using an enzyme immunoassay kit (Enzo Life Science, Plymouth Meeting, PA, ADI-901-065) and assaying individuals in duplicate with a nine-point standard curve, as well as a sample run in triplicate on each plate to calculate inter-assay variation. As with CORT assays, pre- and post-manipulation samples were always run on the same plate. Approximately 2000 cpm of tritiated testosterone was added to each sample to determine sample recovery following two rounds of diethyl ether extraction. Average extraction efficiency was 90.33% for 2015 samples. Because these 2015 average recoveries were consistently high, and the same individual (C. Bergeon Burns) ran testosterone assays both years, we did not quantify extraction efficiency in 2016, in part to maximize plasma volume for the assays. Extracted samples were resuspended in 50 μL of ethanol and diluted in 300 μL of assay buffer. 100 μL quantities were run in duplicate. Inter-assay coefficient of variation for 2015 males was 3.85%, and 4.55% for males in 2016. As with social interactivity and stress-induced CORT data, we analysed changes in testosterone levels (post-manipulation levels – pre-manipulation levels).

### Statistical analysis

We used t-tests to evaluate the overall effect of the plumage colour treatment on measures of interactivity pre- and post-manipulation (change in interactivity with the social mate, with females other than the social mate, and with other males) and a Mann Whitney U test and t-test to examine differences in plumage colour pre- and post-manipulation. Linear models were used to analyse how the degree of change in plumage colour affected changes in social interactivity, changes in testosterone and stress-induced CORT levels, as well as changes in paternity (changes in either extra-pair offspring, proportion of within-pair offspring, or total offspring) in manipulated males. We also considered changes in hormone levels as additional predictor variables in the best model of change in interactivity with the social mate. Similarly, we investigated whether changes in hormone levels predicted changes in interactivity in unmanipulated males. Finally, we investigated whether changes in extra-pair paternity and the proportion of within-pair paternity were related to changes in interactivity. Analyses were done in R v3.3.1^31^.

## Results

Males that were randomly assigned to different treatment groups did not differ in their levels of interactivity prior to the plumage manipulation (all p > 0.40) or in plumage colour (Mann Whitney U test, U = 245, p = 0.48, n = 45 males). Following the plumage manipulation, experimentally manipulated males had darker plumage than unmanipulated males (t-test, t = 4.44, df = 40.98, *Cohen*’*s D* = 1.32, p < 0.0001, n = 45 males, Fig. S1). There was no effect of the plumage colour treatment on interactivity between males and females post-manipulation, including interactions involving just the pair-bonded individuals (t = −1.71, df = 21.52, p = 0.10, n = 14 manipulated males, n = 14 unmanipulated males) or interactions between males and females outside of the pair bond (t = 0.25, df = 30.37, p = 0.80, n = 19 manipulated males, n = 21 unmanipulated males). Similarly, on average, males with darkened plumage were not more interactive with other males (t = −0.76, df = 40.90, p = 0.45, n = 22 manipulated males, n = 22 unmanipulated males).

**Figure 1:**
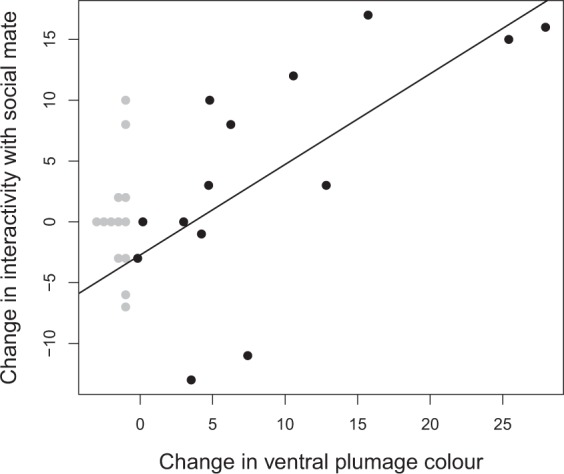
Experimental manipulation of plumage colour affects social interactivity. A male’s change in interactivity with his social mate was strongly predicted by the magnitude of experimental change in ventral plumage colour (β = 0.75, r^2^ = 0.47, p = 0.007, n = 14). Ventral plumage colour was measured as average breast feather brightness and transformed so a larger value corresponded to a darker bird. A greater change in colour was associated with a larger shift from pale to dark. Black points are manipulated males and grey points are unmanipulated males. Grey points are jittered for clarity.

Because male ventral plumage colour is variable, experimentally darkened males experienced different degrees of plumage colour change following the manipulation. Therefore, we would not expect a treatment effect for those males who originally had dark plumage. Among these males with experimentally darkened plumage, changes in social interactivity between males and their social mates were strongly associated with the degree of colour change (β = 0.75, r^2^ = 0.47, p = 0.007, n = 14). Specifically, males who experienced a greater shift in colour (pale to dark) had a larger increase in the number of interactions with their social mate post-manipulation compared to pre-manipulation (Fig. 1).

Change in ventral plumage colour of experimentally darkened males was weakly associated with change in interactivity with females outside of the pair bond (β = 0.23, r^2^ = 0.17, p = 0.04, n = 21, Fig. S2), but not with other males (β = 0.06, r^2^ = −0.03, p = 0.56, n = 22). Although colour change explained a substantial amount of variation in the change in social mate interactivity (r^2^ = 0.47), the model was improved significantly by adding change in testosterone and change in stress-induced CORT as additional predictors (Adj. r^2^ = 0.72). Males who experienced a greater shift in colour (pale to dark), a larger, positive change in testosterone levels, and a dampened stress-induced CORT response had a larger, positive change in the number of interactions with their social mate post-manipulation compared to pre-manipulation (Adj. r^2^ = 0.72, p = 0.01, Table 1, Fig. S3). Testosterone and stress-induced CORT levels were not correlated pre- or post-manipulation; however, change in testosterone and change in stress-induced CORT levels were somewhat correlated (r = 0.57). Therefore, to quantify the relative impact of each predictor, we analysed the average change in r^2^ when adding each predictor to the model across all possible orderings of the predictors (Table 1)^32,33^. This indicates that the change in ventral plumage colour provides the largest contribution in explaining change in social mate interactivity, followed by changes in testosterone and changes in CORT, respectively.

**Table 1:** Predictors of changes in social interactivity between males that had their ventral plumage colour darkened and their social mates (Adj. r^2^ = 0.72, p = 0.01, n = 10).

Predictor of change in social mate interactivity	Coefficient (range of jackknife estimates)^a^	p (range of jackknife values)	*Lmg* ^b^
Change in ventral plumage colour	0.85 (0.75, 0.98)	0.003 (0.003, 0.015)	0.55
Change in testosterone	15.41 (10.15, 20.12)	0.018 (0.015, 0.061)	0.20
Change in stress-induced corticosterone	−0.16 (−0.21, −0.12)	0.047 (0.028, 0.156)	0.07

^a^Jackknife resampling was used to assess robustness of results.

^b^The metric *lmg* estimates the average change in r^2^ when adding each predictor to the model across all possible orderings of the predictors.

**Figure 2:**
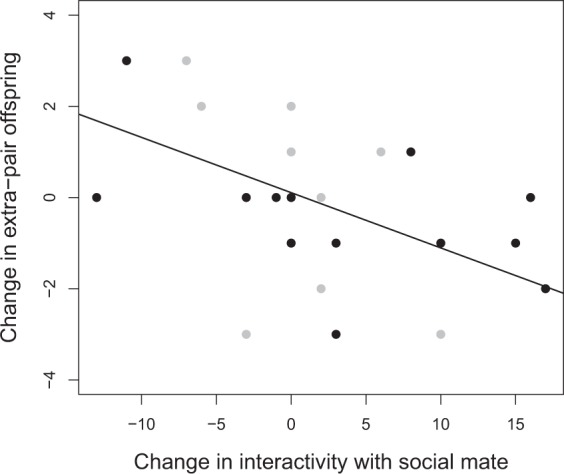
Changes in interactivity within the pair bond affect changes in extra-pair paternity. Males who increased their interactivity with their social mate sired fewer extra-pair offspring across all replacement clutch nests compared to first clutch nests, irrespective of the plumage colour manipulation (β = −0.12, r^2^ = 0.20, p = 0.01, n = 23). Black points are manipulated males and grey points are unmanipulated males.

Similar analysis of unmanipulated males did not show that hormone dynamics explained changes in social interactivity, providing additional evidence for the key role of the ventral plumage colour manipulation in this coordination of the integrated phenotype (T: β = 8.54, CORT: β = 0.11, Adj. r^2^ = 0.35, p = 0.14, n = 8). Changes in paternity (analysed as change in the proportion of within-pair offspring and changes in the number of extra-pair offspring pre- vs. post-manipulation) were not related to change in ventral plumage colour. However, change in the number of extra-pair offspring was negatively related to change in social interactivity with the social mate irrespective of the plumage manipulation. In other words, males who increased their interactivity with their social mate sired fewer extra-pair young across all nests in the replacement clutch compared to eggs collected before start of the experiment (β = −0.12, r^2^ = 0.20, p = 0.01, n = 23) (Fig. 2).

## Discussion

Here, we have overcome two important challenges for studies of social behaviour. First, our behavioural data are based on automated recordings of close-encounter social interactions in an experimental setting. Social interaction data revealed that increased interactivity with females – especially the social mate – and not competing males is influenced by a signal trait manipulation. Second, by experimentally manipulating a socially relevant signal trait and quantifying changes in circulating hormones and reproductive success, we provide evidence for dynamic coordination and feedback among an individual’s degree of signal trait elaboration, behaviour, physiological state, and success siring extra-pair offspring. Changes in ventral plumage colour and changes in testosterone and stress-induced CORT together explain a substantial amount of variation in changes in interactivity within social pairs over the course of one week. This provides evidence that females respond to their mates, and in turn, influence their mate’s physiology.

In most studies of wild populations, temporal variation in hormone levels within individuals is either not sampled or regarded as noise or laboratory error^34,35^. However, when we account for the changes in ventral plumage colour, there are corresponding positive changes in testosterone levels and negative changes in stress-induced CORT levels within individuals that explain additional variation in changes in social interactivity during the two sampling points. Previous research in this system demonstrated that male testosterone levels responded to plumage colour manipulation^9^, but the mechanism was not yet known. Our work here suggests that behavioural changes in response to the plumage manipulation influence hormone levels, which further drives changes in social interactivity. Experimental studies have previously shown behaviourally-mediated changes in hormones^8^ and therefore we hypothesize that females alter their behaviour towards their manipulated mates, which drives changes in male hormone levels, and further contributes to changes in behaviour between the mated pair. Male-male interactions did not change in predictable ways in response to the phenotype manipulation.

A larger, negative change in stress-induced CORT response was one of the predictors of a greater, positive change in a male’s social interactivity with his mate. Males with greater increases in interactivity had lower stress-induced CORT in their post-manipulation sample compared to their pre-manipulation sample. Increased CORT has been shown to suppress testosterone levels^36^; however, testosterone implantation studies show some evidence for corresponding increases in CORT^37^. A greater amount of circulating CORT in response to stress is generally considered adaptive, as this hormone can quickly induce the emergency life history state^38^. The dampened stress-induced CORT response reported here could suggest that individuals pushed beyond their normal behavioural and testosterone optima cannot cope as well with the stress challenge. Because we lack baseline (immediately after capture) CORT measures, it is possible that changes in baseline CORT resulted in less or no change in the scope of the stress response if increased social interactions within the pair bond lowers baseline CORT.

Experimental changes in plumage colour did not predict changes in reproductive success, as was found in a previous study of this system^15^. A possible explanation is that breeding was synchronized via egg collection in the current study but not in Safran *et al*.^15^, and this could have altered reproductive behaviour. Males who increased their interactions with their social mate during the post-manipulation data collection period compared to the pre-manipulation period sired fewer extra-pair offspring outside the pairbond in the replacement clutch compared to their first (collected) clutch. This suggests that there is a trade-off between interacting with the social mate – and this could include mate guarding – and seeking extra-pair copulations. However, we did not find a corresponding increase in within-pair paternity in males who increased their interactivity with their social mates.

## Conclusions

This work provides novel experimental evidence that social and reproductive behaviour and glucocorticoid and androgen hormone levels reorganize when the animal’s morphological phenotype is altered. Social behaviour has long been assumed as an important mediator of phenotype integration, but until now, we did not understand the degree to which social interactions are sensitive to cascading changes in the hormonal and morphological phenotype.

## Electronic supplementary material

Supplemental dataset

## Data Availability

All data generated or analysed during this study are available from the corresponding author on reasonable request.
