# Supplementary material for: Experimental manipulation of a signal trait reveals complex phenotype-behaviour coordination
Source: Sci Rep. 2018 Oct 19;8:15533. doi: 10.1038/s41598-018-33948-0 (PMC6195576; doi:10.1038/s41598-018-33948-0)
Supplement: Supplementary file 1 — Supplemental dataset [file 41598_2018_33948_MOESM1_ESM.docx]

Experimental manipulation of a signal trait reveals complex phenotype-behaviour coordination

Iris I. Levin, Bailey K. Fosdick, Toshi Tsunekage, Matthew A. Aberle, Christine M. Bergeon-Burns, Amanda K. Hund, Rebecca J. Safran

**Figure S1:** Boxplot illustrating that experimental darkening of male ventral plumage colour resulted in lower average breast brightness in manipulated barn swallow males compared to unmanipulated males (t-test, t=4.44, df=40.98, *Cohen’s D*=1.32, p<0.0001*,* n=45 males). A lower average breast brightness corresponds to an individual with darker ventral plumage.

**

**Figure S2:** Change in ventral plumage colour was related to changes in interactivity with females other than the male’s social mate (β=0.23, r^2^=0.17, p=0.04, n=21).

**Figure S3:** Conditional plots for visualizing the three significant predictors of change in a male’s interactivity with his social mate. These predictors are A. change in ventral plumage colour, B. change in testosterone, and C. change in stress-induced corticosterone. Conditional plots were made in the R package *visreg*^1^ and use the median for the other two terms in the model.

*References*

^1^Breheny P, Burchett W. 2013. Visualizing regression models using visreg. http://myweb.uiowa.edu/pbreheny/publications/visreg.pdf.
